# Supplementary material for: Radial glia integrin avb8 regulates cell autonomous microglial TGFβ1 signaling that is necessary for microglial identity
Source: Nat Commun. 2025 Mar 22;16:2840. doi: 10.1038/s41467-025-57684-y (PMC11929771; doi:10.1038/s41467-025-57684-y)
Supplement: Supplementary file 1 — Supplementary Information [file 41467_2025_57684_MOESM1_ESM.pdf]

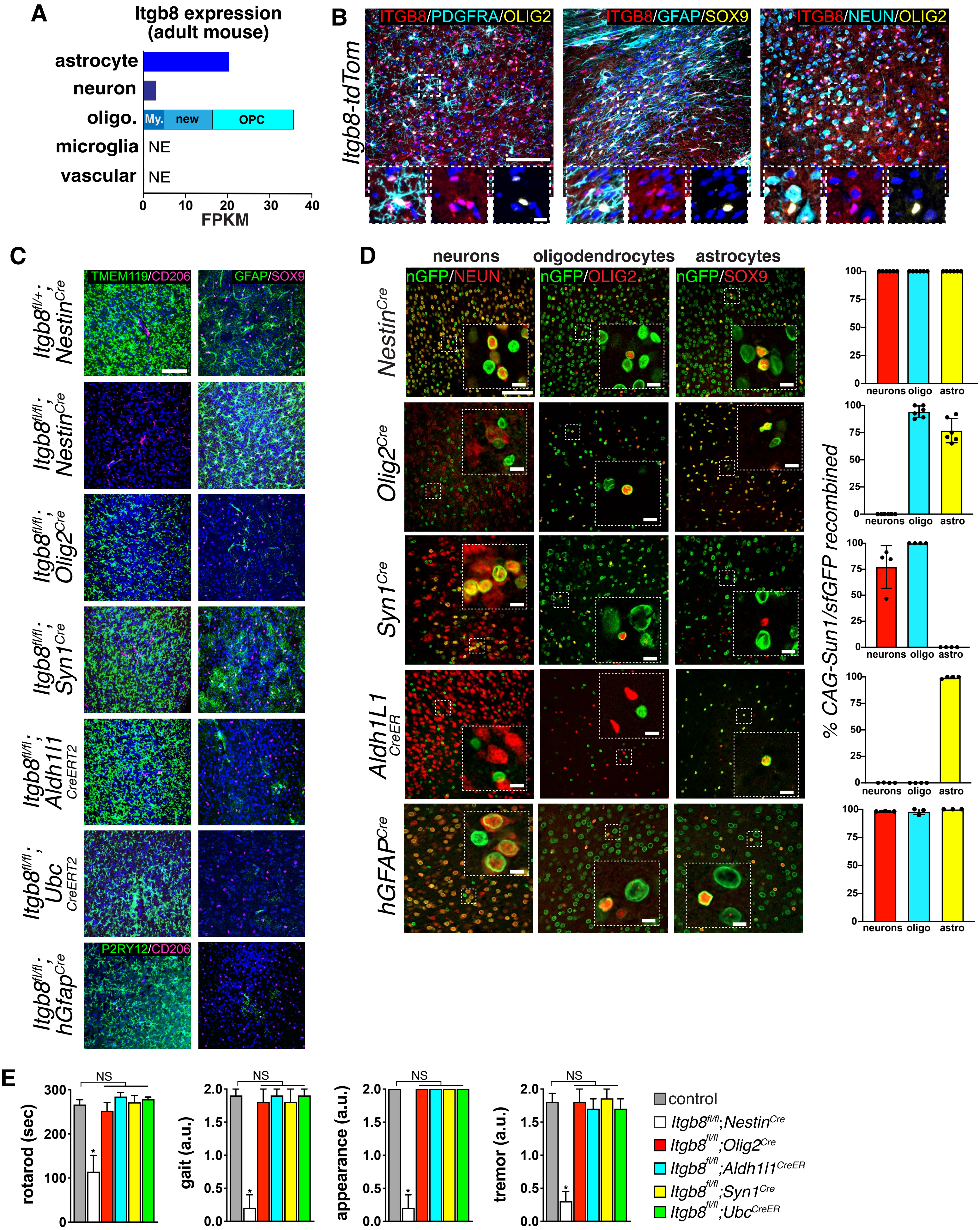
A
Itgb8 expression (adult mouse)
astrocyte
neuron
oligo.
My.
new
OPC
microglia
NE
vascular
NE
FPKM
B
Itgb8-tdTom
ITGB8/PDGFRA/OLIG2
ITGB8/GFAP/SOX9
ITGB8/NEUN/OLIG2
C
TMEM119/CD206
GFAP/SOX9
P2RY12/CD206
Itgb8fl/+; NestinCre
Itgb8fl/fl; NestinCre
Itgb8fl/fl; Olig2Cre
Itgb8fl/fl; Syn1Cre
Itgb8fl/fl; Aldh1l1 CreERT2
Itgb8fl/fl; Ubc CreERT2
Itgb8fl/fl; hGfapCre
D
neurons
oligodendrocytes
astrocytes
nGFP/NEUN
nGFP/OLIG2
nGFP/SOX9
NestinCre
Olig2Cre
Syn1Cre
Aldh1L1 CreER
hGFAPCre
% CAG-Sun1/sfGFP recombined
neurons
oligo
astro
E
rotarod (sec)
gait (a.u.)
appearance (a.u.)
tremor (a.u.)
NS
control
Itgb8fl/fl;NestinCre
Itgb8fl/fl;Olig2Cre
Itgb8fl/fl;Aldh1l1CreER
Itgb8fl/fl;Syn1Cre
Itgb8fl/fl;UbcCreER

Supplemental Figure 1. Deletion of *Itgb8* in adulthood does not disrupt microglial homeostasis.

A) Expression analysis of *Itgb8* expression in adult astrocytes, neurons, oligodendrocytes, microglia, and endothelial cells[35] (FPKM=Fragments per kilobase of transcript per million mapped reads). B) *Itgb8* is expressed in oligodendrocytes ($OLIG2^{+}$; $PDGFRA^{+}$), OPCs ($OLIG2^{+}$), astrocytes (SOX9, $GFAP^{+/-}$), and neurons ($NEUN^{+}$) in adulthood, as shown by immunohistochemical overlap with a *Itgb8-TdT* reporter line. C) Deletion of *Itgb8* using various cell-lineage restricted *Cre* and *CreER* mouse lines does not result in changes in microglial homeostasis or associated astrocyte activation, unlike deletion with $\textit{Nestin}^{\textit{Cre}}$. D) Cre-dependent nuclear GFP *Cre*-reporter line *SunTag* crossed to indicated *Cre* or *CreER* line, and brain sections from adult mice were stained for nuclear-localized markers of neurons (NEUN), oligodendrocytes and oligodendrocyte precursors (OPCs) (OLIG2), or astrocytes (SOX9); images taken from motor cortex. Cell marker colocalization with *CAG-Sun1/sfGFP* recombination to the right. 6 animals were quantified for $\textit{Nestin}^{\textit{Cre}}$ and $\textit{Olig2}^{\textit{Cre}}$, 4 for $\textit{Syn1}^{\textit{Cre}}$ and $\textit{Aldh1L1}^{\textit{CreER}}$ and 3 for $\textit{hGFAP}^{\textit{Cre}}$. 3 images per mouse were quantified from the cerebral cortex. E) Deletion of *Itgb8* using various cell-lineage restricted *Cre* and *CreER* mouse lines does not result in changes in motor behavior, unlike deletion with $\textit{Nestin}^{\textit{Cre}}$, which results in profound motor dysfunction. n=5 per genotype. Error bars= mean +/- SEM. Scale bars in B,C and D=100µm, inset scale bar=10µm. Source data are provided as a Source Data file.

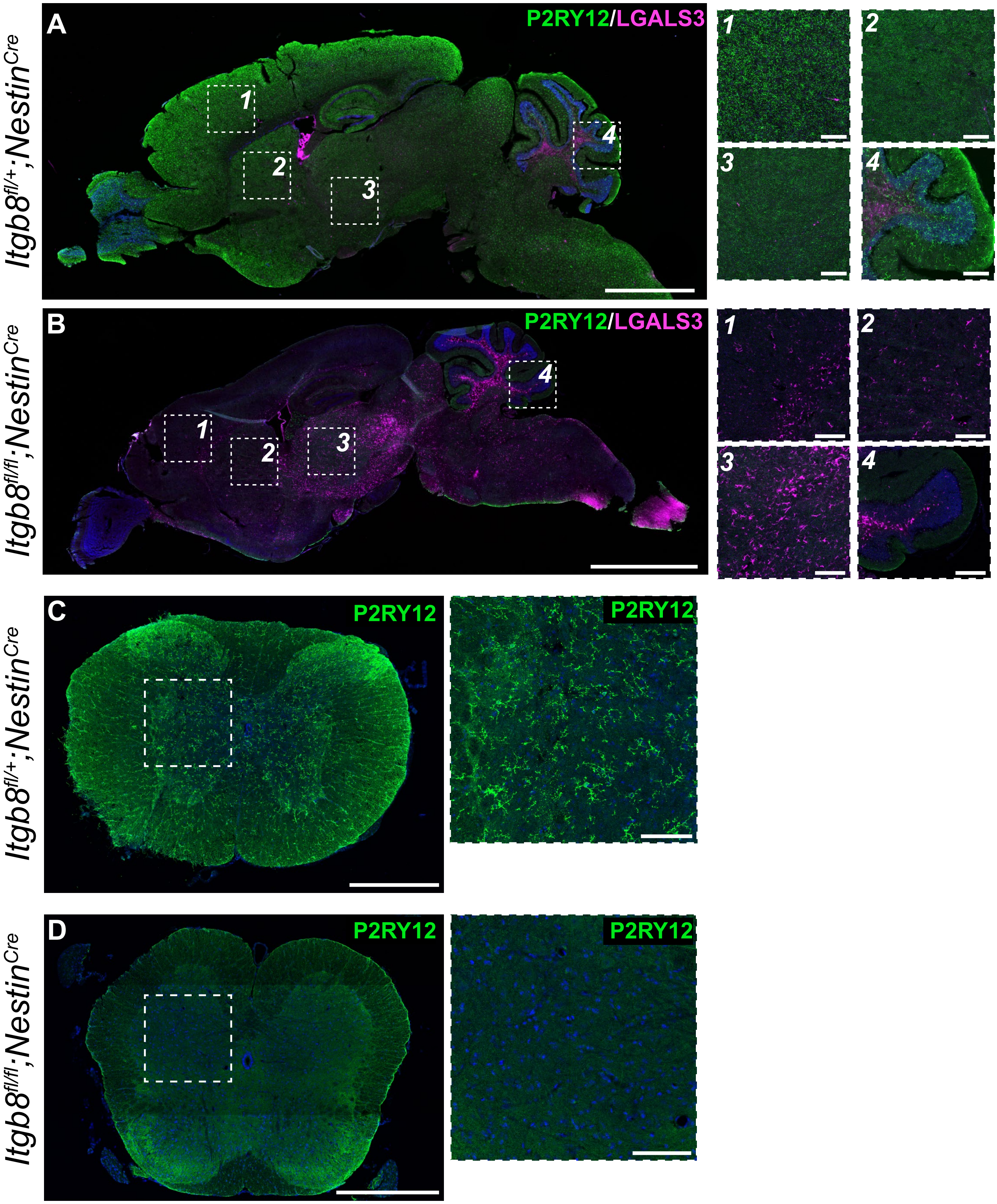

Supplemental Figure 2. CNS-wide microglial phenotype in *Itgb8$^{fl/fl}$;Nestin^{Cre}* mutants.

A,B) Brain-wide deletion of *Itgb8* with *Nestin^{Cre}* results in loss microglial of microglial homeostasis and increased reactivity, as shown by loss of P2RY12 expression and upregulation of the reactive marker LGALS3. Blow-ups in 1-4 highlight protein expression changes in the cerebral cortex, striatum, thalamus, and cerebellum respectively. C, D) Loss of *Itgb8* expression by deletion with *Nestin^{Cre}* also results in the loss of microglial homeostasis in the spinal cord, as shown by loss of P2RY12 expression. Scale bar in A,B=1mm and insets are 100µm. Scale bar in C,D= 250µm, insets are 50µm.

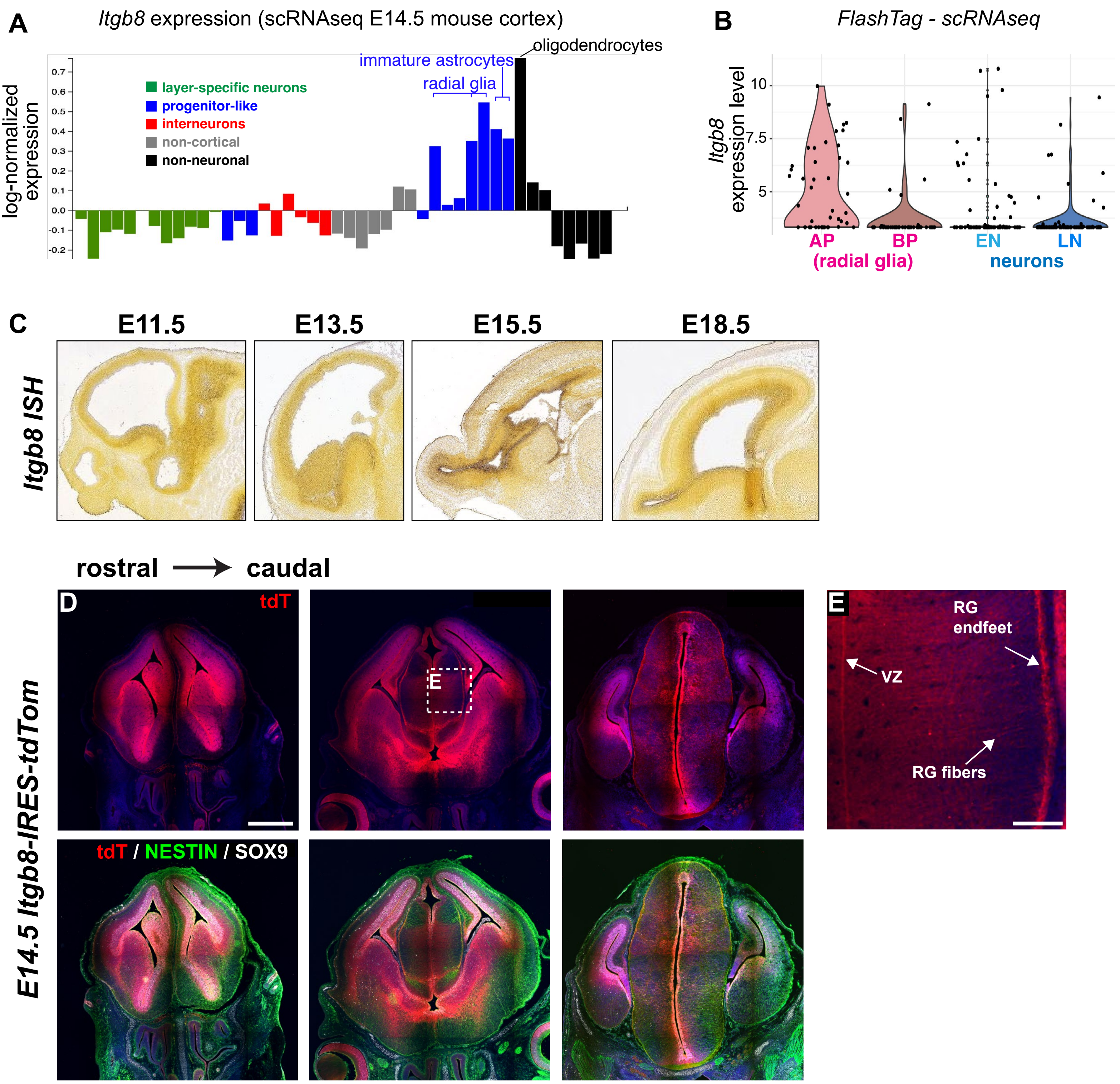

Supplemental Figure 3. Analysis of cell-type specific *Itgb8* expression.

A) Single-cell RNA-seq analysis of *Itgb8* expression in the developing brain, from[17]maturation, and migration events, ultimately giving rise to a wide variety of neuronal and non-neuronal cell types. To better understand cellular and molecular processes that unfold during late corticogenesis, we perform single-cell RNA-seq on the mouse cerebral cortex at a progenitor driven phase (embryonic day 14.5. Individual columns represent clustered categories of cell types. B) *Itgb8* expression in apical and basal progenitors, early born and late born neurons, derived from Flash-Tag ScRNA-seq analysis in [18]. C) *Itgb8* in situ hybridization (ISH) at indicated time points taken from Allen Brain Atlas (https://mouse.brain-map.org). D) Analysis of *Itgb8*^{tdT} expression in E14.5 mouse brain stained for radial glia nuclei (SOX9, white) and radial glia processes (NESTIN, green). E) Magnified image of the embryonic thalamus from (D), showing *Itgb8*^{tdT} expression in the ventricular progenitor zone, radial glia fibers and radial glia endfeet in the overlying meninges. Scale bar in D=500μm, inset is 100μm.

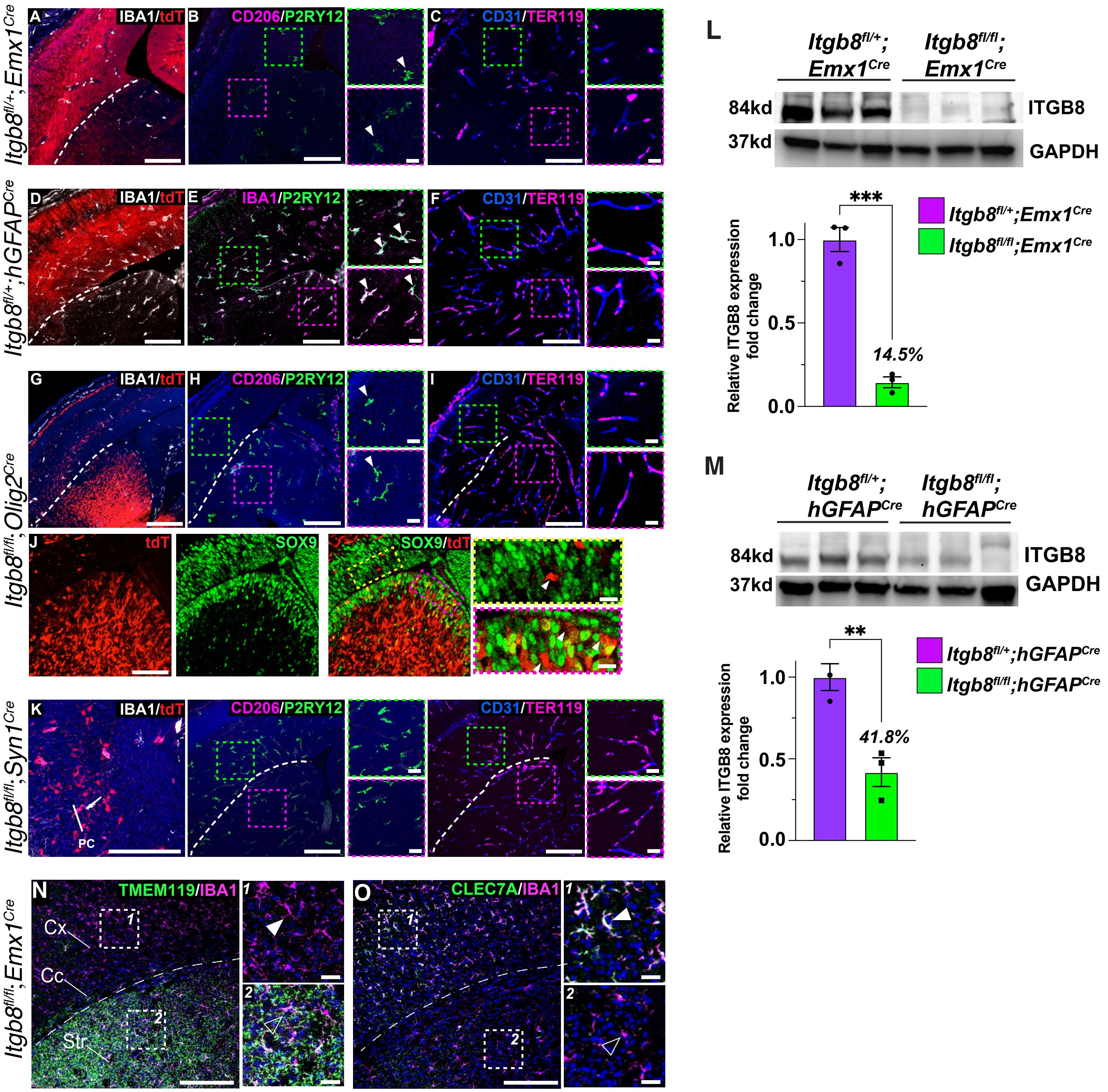

Itgb8fl/+;Emx1Cre
A IBA1/tdT
B CD206/P2RY12
C CD31/TER119
Itgb8fl/+;hGFAPCre
D IBA1/tdT
E IBA1/P2RY12
F CD31/TER119
Itgb8fl/fl;Olig2Cre
G IBA1/tdT
H CD206/P2RY12
I CD31/TER119
J tdT
SOX9
SOX9/tdT
Itgb8fl/fl;Syn1Cre
K IBA1/tdT
PC
CD206/P2RY12
CD31/TER119
L
Itgb8fl/+; Emx1Cre
Itgb8fl/fl; Emx1Cre
84kd
37kd
ITGB8
GAPDH
***
Relative ITGB8 expression fold change
1.0
0.5
0.0
14.5%
Itgb8fl/+;Emx1Cre
Itgb8fl/fl;Emx1Cre
M
Itgb8fl/+; hGFAPCre
Itgb8fl/fl; hGFAPCre
84kd
37kd
ITGB8
GAPDH
**
Relative ITGB8 expression fold change
1.0
0.5
0.0
41.8%
Itgb8fl/+;hGFAPCre
Itgb8fl/fl;hGFAPCre
Itgb8fl/fl;Emx1Cre
N TMEM119/IBA1
Cx
Cc
Str
1
2
O CLEC7A/IBA1
1
2

Supplemental Figure 4. Cre recombination and phenotypes in *Itgb8* conditional mutants.

A) Coronal brain sections from E14.5 $Itgb8^{fl/+};Emx1^{Cre}$ control embryos stained for tdT (Cre recombination, red) and microglia/macrophages (IBA1, white); B) microglia (P2RY12 in green, see arrowheads) and immature macrophages (CD206 in magenta) and C) hemorrhage (red blood cells marked by TER119 in magenta and vascular endothelium marked by CD31 in blue). D) Coronal brain sections from E14.5 $Itgb8^{fl/+};hGFAP^{Cre}$ control embryos stained for tdT (Cre recombination, red) and microglia/macrophages (IBA1, white); E) microglia (P2RY12 in green, IBA1 in magenta, see arrowheads) and F) hemorrhage (red blood cells marked by TER119 in magenta and vascular endothelium marked by CD31 in blue). G) E14.5 $Itgb8^{fl/fl};Olig2^{Cre}$ mice stained for tdT (Cre recombination, red) and microglia/macrophages (IBA1, white); H) microglia (P2RY12 in green, see arrowheads) and immature macrophages (CD206 in magenta); I) hemorrhage (red blood cells marked by TER119 in magenta and vascular endothelium marked by CD31 in blue); J) tdT (red) and apical progenitors (SOX9, green). Note lack of recombination of apical progenitors and also lack of microglia or vascular/hemorrhage phenotypes in these mice. K) Coronal brain sections from E14.5 $Itgb8^{fl/fl};Syn1^{Cre}$ showing expression of tdT (Cre recombination, red) and microglia (P2RY12 in green, see arrowheads) and immature macrophages (CD206 in magenta); hemorrhage (red blood cells marked by TER119 in magenta and vascular endothelium marked by CD31 in blue). L) Western blot analysis of ITGB8 protein expression in E14.5 $Itgb8^{fl/+};Emx1^{Cre}$ and $Itgb8^{fl/-fl};Emx1^{Cre}$ embryonic cerebral cortex. M) Western blot analysis of Itgb8 protein expression in E14.5 $Itgb8^{fl/+};hGFAP^{Cre}$ and $Itgb8^{fl/fl};hGFAP^{Cre}$ embryonic cerebral cortex. n=3 per genotype. N) Downregulation of the microglial homeostatic marker TMEM119 in the cortex of a $Itgb8^{fl/fl}; Emx1^{Cre}$ mouse, and maintenance in the underlying striatum. O) Cortex-restricted upregulation of the microglial reactive marker CLEC7A in IBA1^{+} cells of the cerebral cortex, and lack of CLEC7A expression in microglia in the underlying striatum. Closed and open arrowheads in N and O mark dysmature cortical and phenotypically normal microglia respectively. Error bars= mean +/- SEM. Cx=cerebral cortex, Cc= corpus callosum. Scale bar in A-K=150μm, inset scale bars= 25μm. Scale bar in N,O=150μm and inset bars=20μm. ****p<.0001, **p<.01, two-tailed unpaired t-test. Source data are provided as a Source Data file.

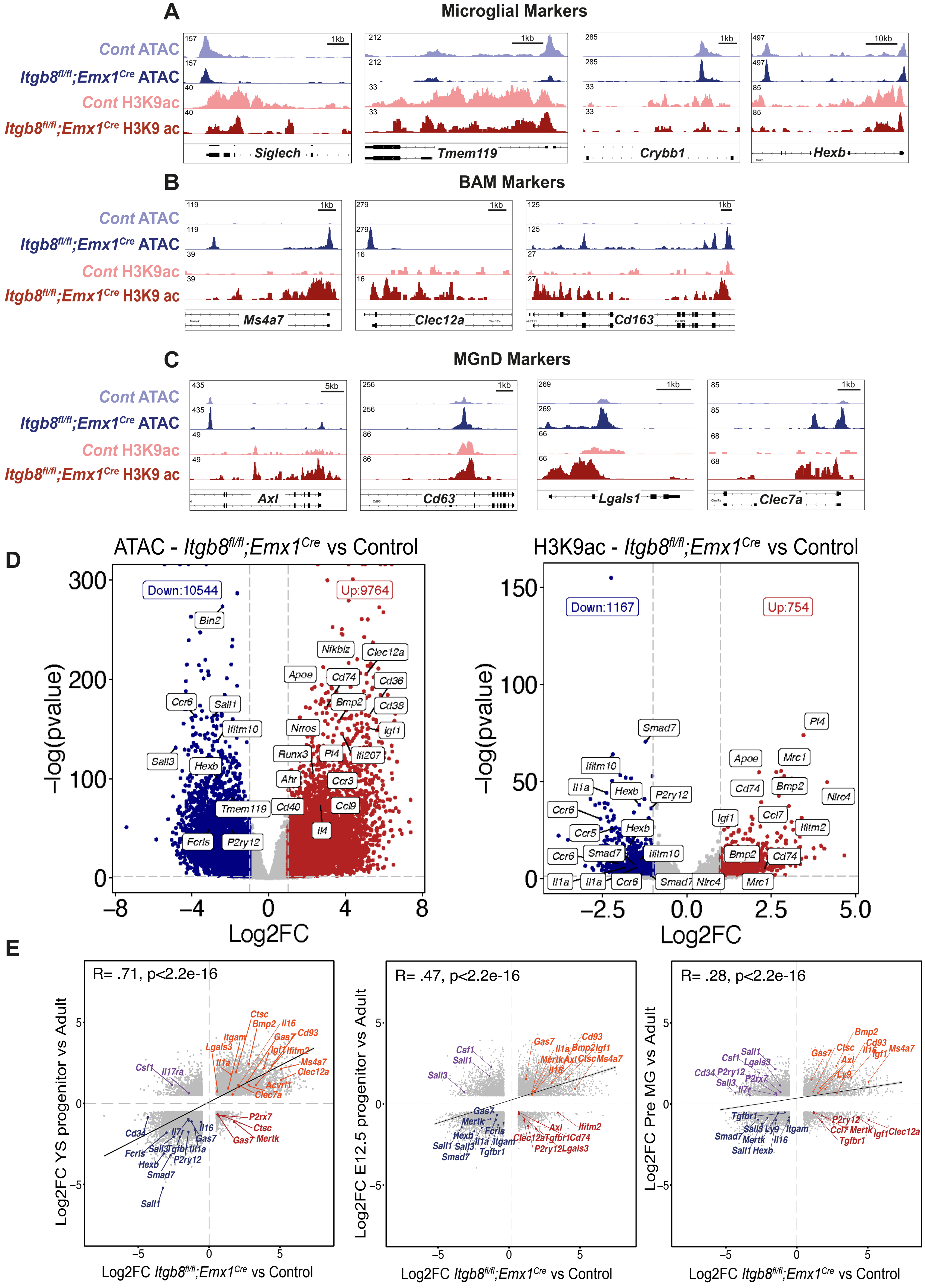
A
Microglial Markers
Cont ATAC
Itgb8fl/fl;Emx1Cre ATAC
Cont H3K9ac
Itgb8fl/fl;Emx1Cre H3K9 ac
Siglech
Tmem119
Crybb1
Hexb
1kb
10kb
B
BAM Markers
Ms4a7
Clec12a
Cd163
C
MGnD Markers
Axl
Cd63
Lgals1
Clec7a
5kb
D
ATAC - Itgb8fl/fl;Emx1Cre vs Control
Down:10544
Up:9764
H3K9ac - Itgb8fl/fl;Emx1Cre vs Control
Down:1167
Up:754
−log(pvalue)
Log2FC
E
R= .71, p<2.2e-16
R= .47, p<2.2e-16
R= .28, p<2.2e-16
Log2FC YS progenitor vs Adult
Log2FC E12.5 progenitor vs Adult
Log2FC Pre MG vs Adult
Log2FC Itgb8fl/fl;Emx1Cre vs Control

Supplemental Figure 5. *Itgb8* deletion results in epigenetic changes in BAM and disease-associated macrophage gene bodies.

A-C) Genome browser profiles for and *Itgb8*$^{fl/fl}$ control and *Itgb8*$^{fl/fl}$*;Emx1*^{Cre} ATAC-seq chromatin accessibility and histone H3K9ac ChIP-seq enrichment. Noted are prominent markers of A) microglia, B) borderzone macrophages and C) neurodegenerative disease associated (MGnD) microglia. D) Volcano plot analysis of genome-wide *Itgb8*$^{fl/fl}$ control and *Itgb8*$^{fl/fl}$*;Emx1*^{Cre} ATAC-seq chromatin accessibility (n=7 control, 7 mutant) and histone H3K9ac ChIP-seq enrichment (n=4 control, 3 mutant). E) Correlation plots of *Itgb8*$^{fl/fl}$*;Emx1*^{Cre} ATAC-seq chromatin accessibility with ATAC-seq analysis derived from 1) yolk sac microglial progenitors, 2) E12.5 immature microglia and 3) postnatal day 3 microglia (Matcovitch-Natan et al., 2016).

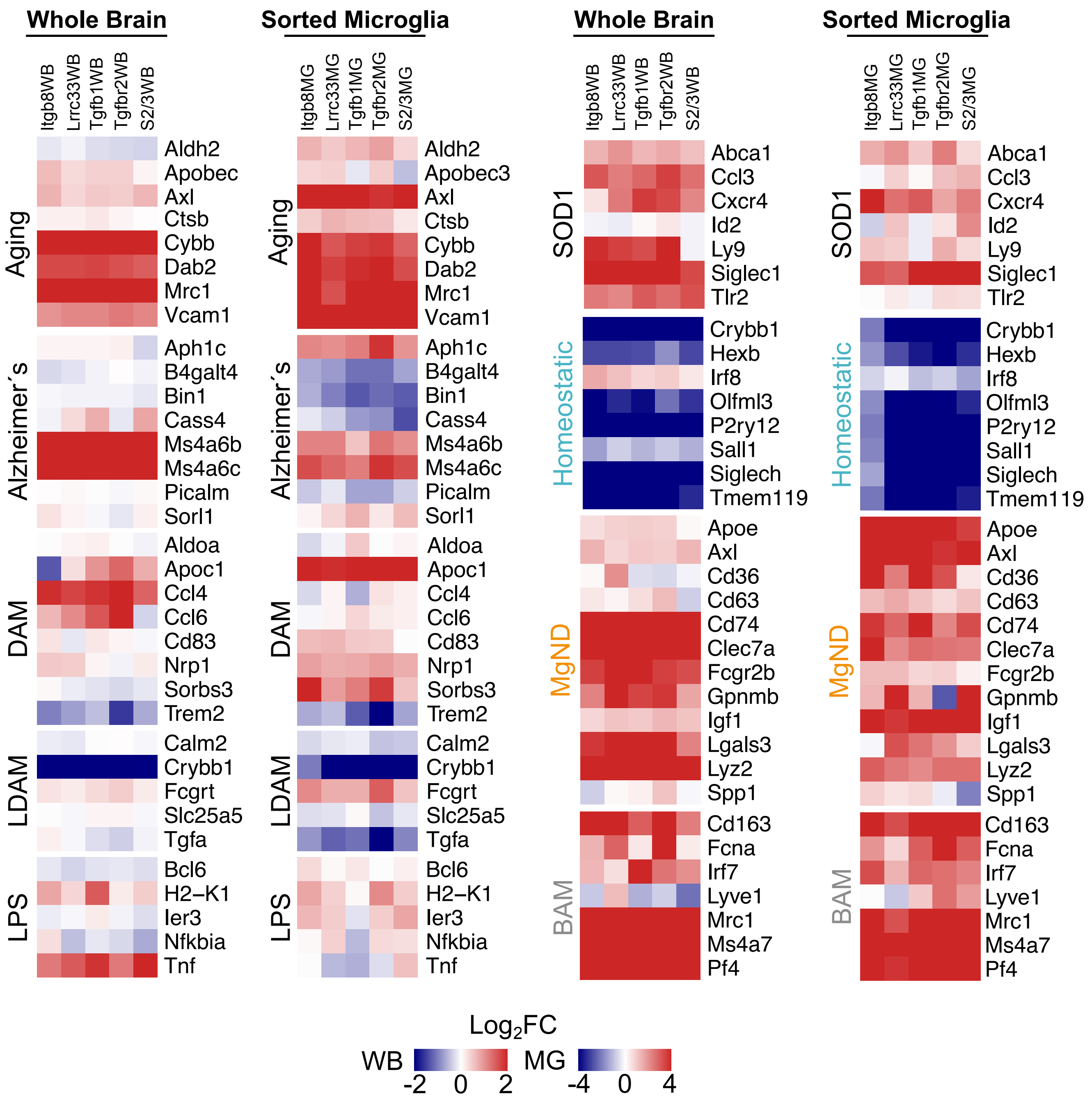

Supplemental Figure 6. Transcriptional analysis of whole brain and FACS isolated microglia in different TGFβ mutant models.

Comparative gene expression analysis in *Itgb8*, *Lrrc33*, *Tgfb1*, *Tgfbr2 and Smad2/3* conditional mutant mouse models. RNA derived from whole brain (WB) and sorted microglia (MG) were compared, focusing on disease and injury associated transcripts.

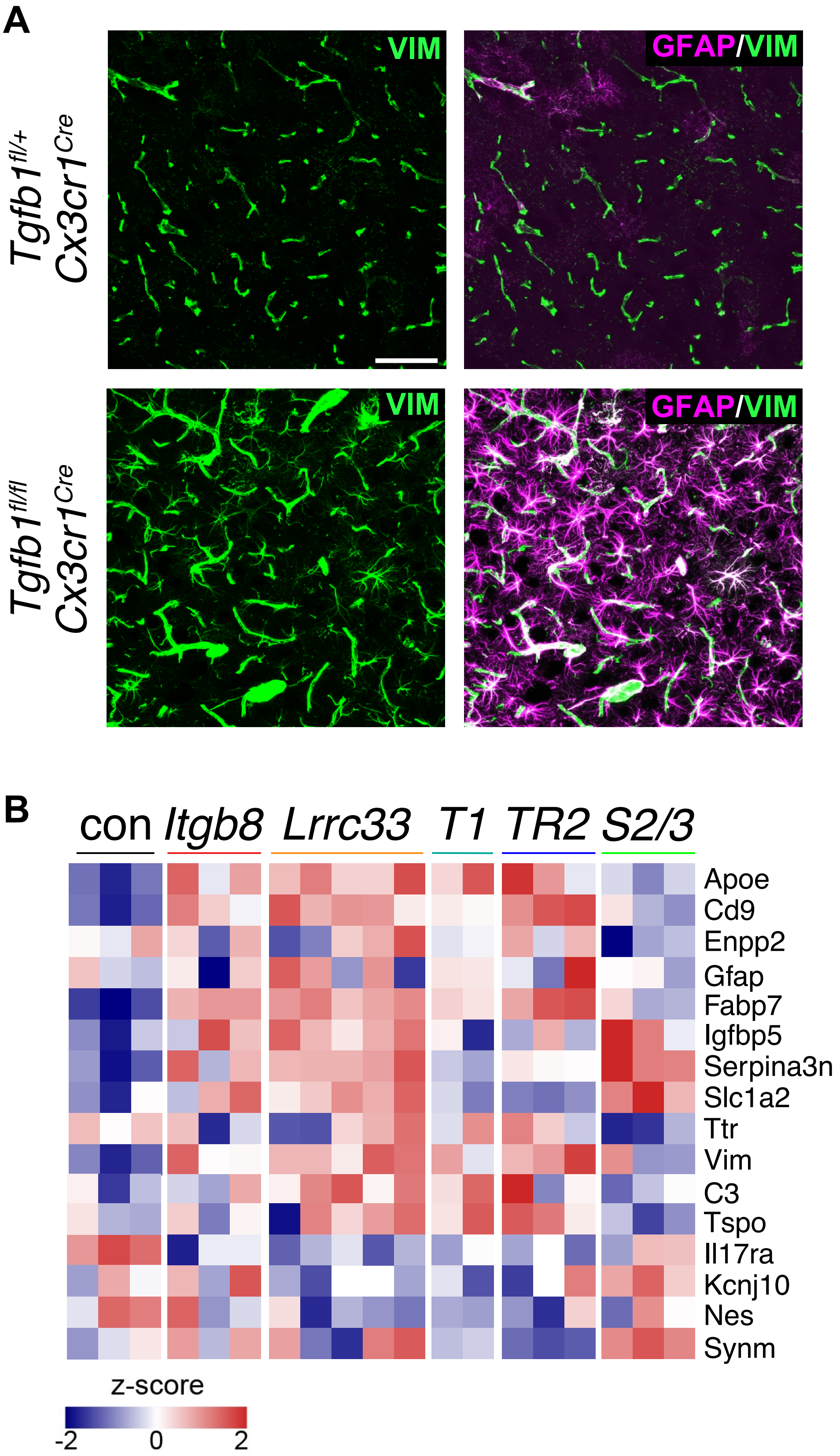

Supplemental Figure 7. Astrocytosis related gene expression in TGFβ pathway mutants.

A) Upregulation of astrocytosis-related genes GFAP and VIM in *Tgfb1*$^{fl/fl}$;*Cx3cr1*^{Cre} mice. B) Comparative gene expression analysis of astrocytosis markers in various TGFβ pathway mutants reveals increased expression of astrocytosis markers in *Itgb8*, *Lrrc33*, *Tgfb1*, *Tgfbr2* conditional mutant models. In *Smad2/3* conditional mutants however, the astrocytosis markers *Apoe, Gfap*, *Fabp7* and *Vim* were not as highly upregulated. Scale bar in A=100 µm.

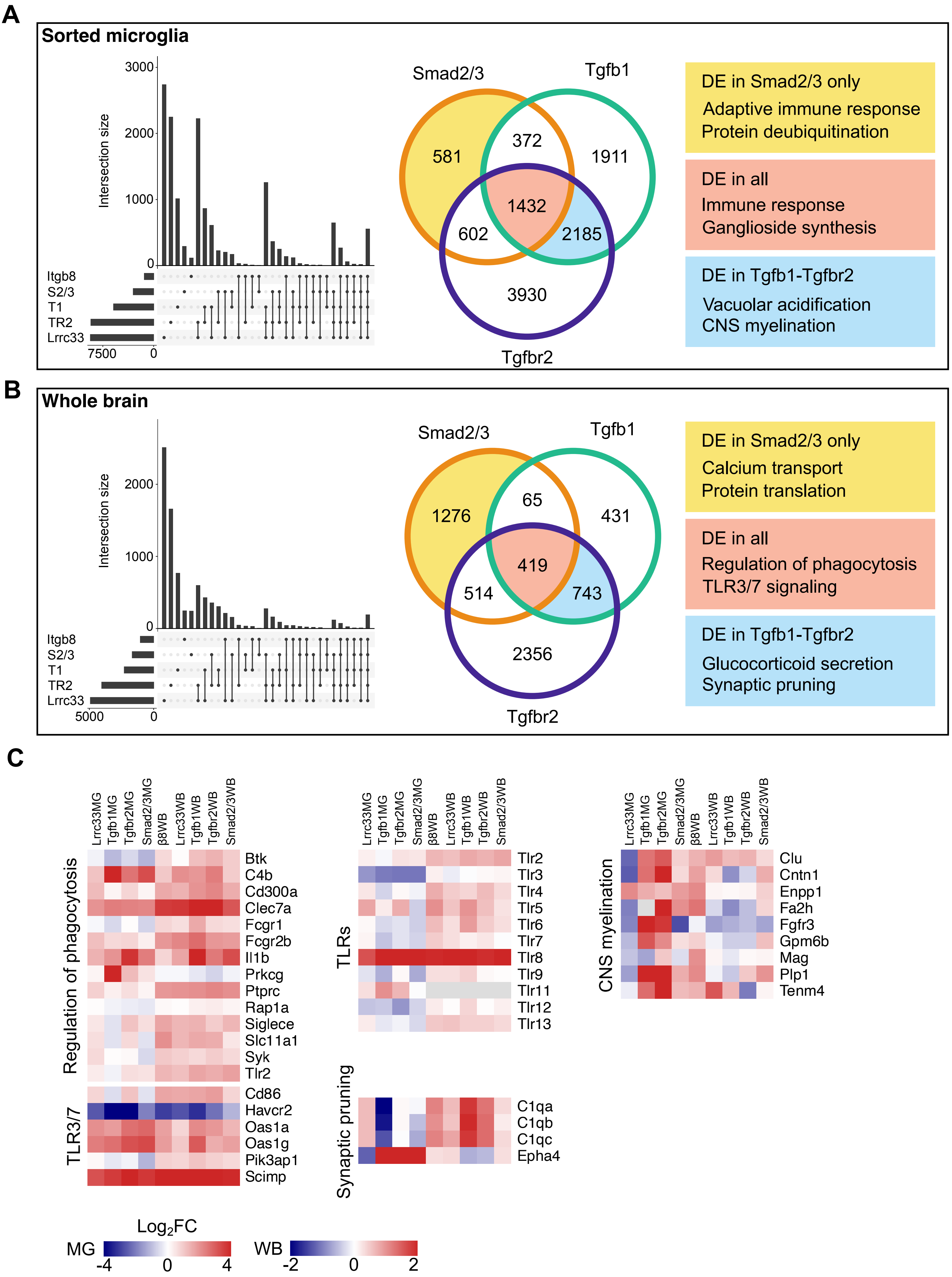
A
Sorted microglia
Intersection size
3000
2000
1000
0
Itgb8
S2/3
T1
TR2
Lrrc33
7500
0
Smad2/3
Tgfb1
Tgfbr2
581
372
1911
1432
602
2185
3930
DE in Smad2/3 only
Adaptive immune response
Protein deubiquitination
DE in all
Immune response
Ganglioside synthesis
DE in Tgfb1-Tgfbr2
Vacuolar acidification
CNS myelination
B
Whole brain
Intersection size
2000
1000
0
Itgb8
S2/3
T1
TR2
Lrrc33
5000
0
Smad2/3
Tgfb1
Tgfbr2
1276
65
431
419
514
743
2356
DE in Smad2/3 only
Calcium transport
Protein translation
DE in all
Regulation of phagocytosis
TLR3/7 signaling
DE in Tgfb1-Tgfbr2
Glucocorticoid secretion
Synaptic pruning
C
Lrrc33MG
Tgfb1MG
Tgfbr2MG
Smad2/3MG
β8WB
Lrrc33WB
Tgfb1WB
Tgfbr2WB
Smad2/3WB
Regulation of phagocytosis
Btk
C4b
Cd300a
Clec7a
Fcgr1
Fcgr2b
Il1b
Prkcg
Ptprc
Rap1a
Siglece
Slc11a1
Syk
Tlr2
TLR3/7
Cd86
Havcr2
Oas1a
Oas1g
Pik3ap1
Scimp
TLRs
Tlr2
Tlr3
Tlr4
Tlr5
Tlr6
Tlr7
Tlr8
Tlr9
Tlr11
Tlr12
Tlr13
Synaptic pruning
C1qa
C1qb
C1qc
Epha4
CNS myelination
Clu
Cntn1
Enpp1
Fa2h
Fgfr3
Gpm6b
Mag
Plp1
Tenm4
Log2FC
MG
-4
0
4
WB
-2
0
2

Supplemental Figure 8. Gene ontology and transcriptional analysis of TGFβ mutant models.

A-B) Upset plots and gene ontology analysis of A) sorted microglia and B) bulk-Seq from different TGFβ mutant models. Putative genes involved in differential neuromotor phenotypes in TGFβ mutants were selected based on differential expression in distinct mouse models. Overrepresentation analysis was performed in each set separately as indicated by respective colors. C) Expression changes of genes driving enrichment of selected GO terms. Levels are shown as Log_2FoldChange from sorted microglia and whole brain from all analyzed mutants. We selected processes likely to be important in microglia-associated neuromotor impairments (i.e. regulation of phagocytosis, Toll-like receptor gene family and signaling, synaptic pruning, and myelination). Color scales are distinct for microglia and whole brain.

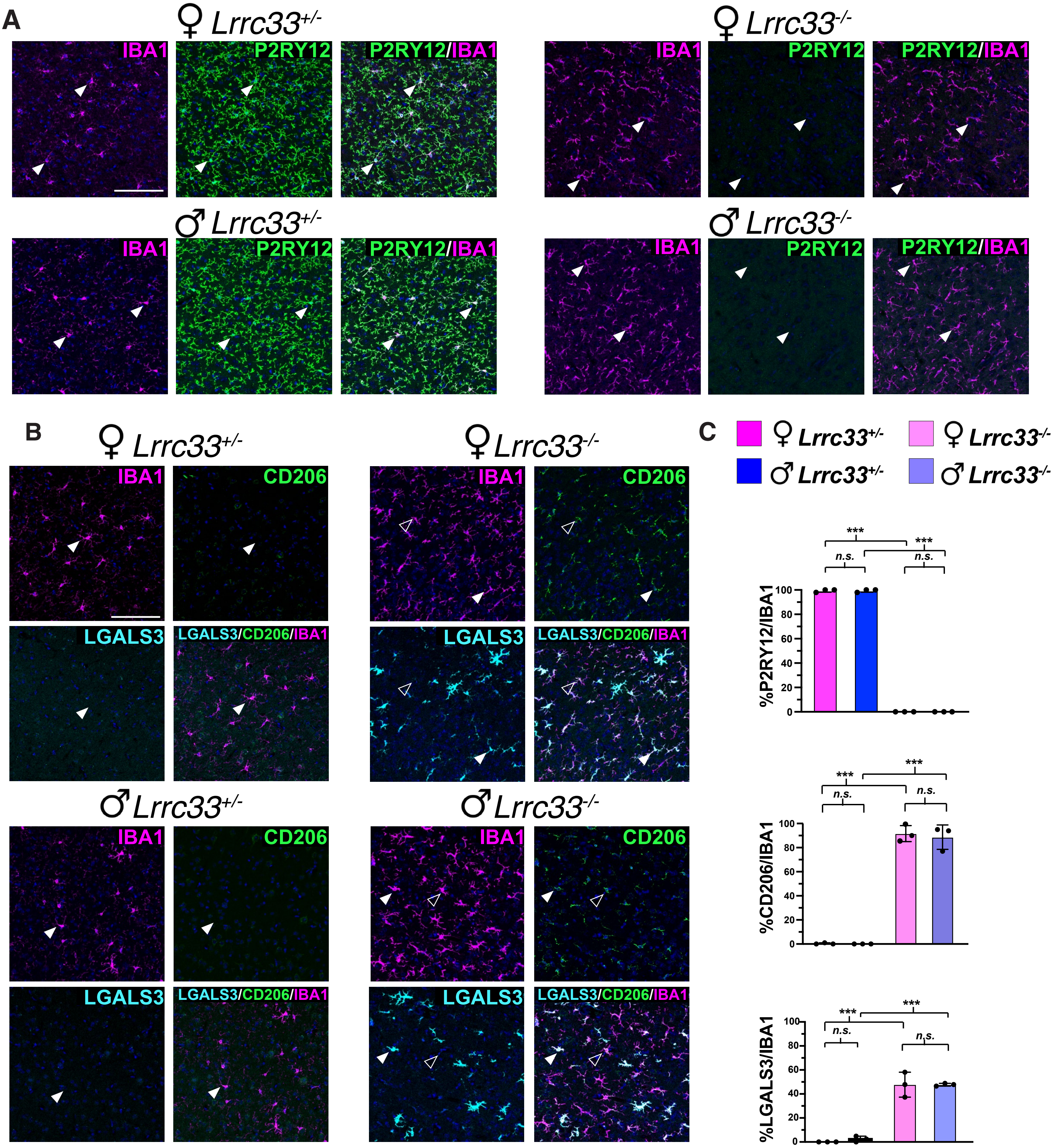

Supplemental Figure 9. Analysis of microglial phenotype in male and female *Lrrc33* mutant mice.

A) Representative images of P60 *Lrrc33*$^{+/-}$ (left) and *Lrrc33*$^{-/-}$ (right) animals immunostained for IBA1 and P2RY12. Top row, images from female mice. Bottom row, images from male mice. Closed arrowhead: microglia. B) Representative images of P60 *Lrrc33*$^{+/-}$ (left) and *Lrrc33*$^{-/-}$ (right) animals immunostained for IBA1, CD206, and LGALS3. Top row, images from female mice. Bottom row, images from male mice. Closed arrowhead: microglia. Some microglia were CD206/IBA1 positive and LGALS3 negative (open arrowhead). C) Quantification of *Lrrc33*$^{+/-}$ and *Lrrc33*$^{-/-}$ image sets shown in A and B. For quantification in C, n=3: 3 images were used from each mouse; 3 mice were analyzed per genotype. Error bars= mean +/- SEM. Scale bars in A,B= 100µm. ***p<0.001, two-tailed unpaired t-test, *n.s.*= not significant. Source data are provided as a Source Data file.

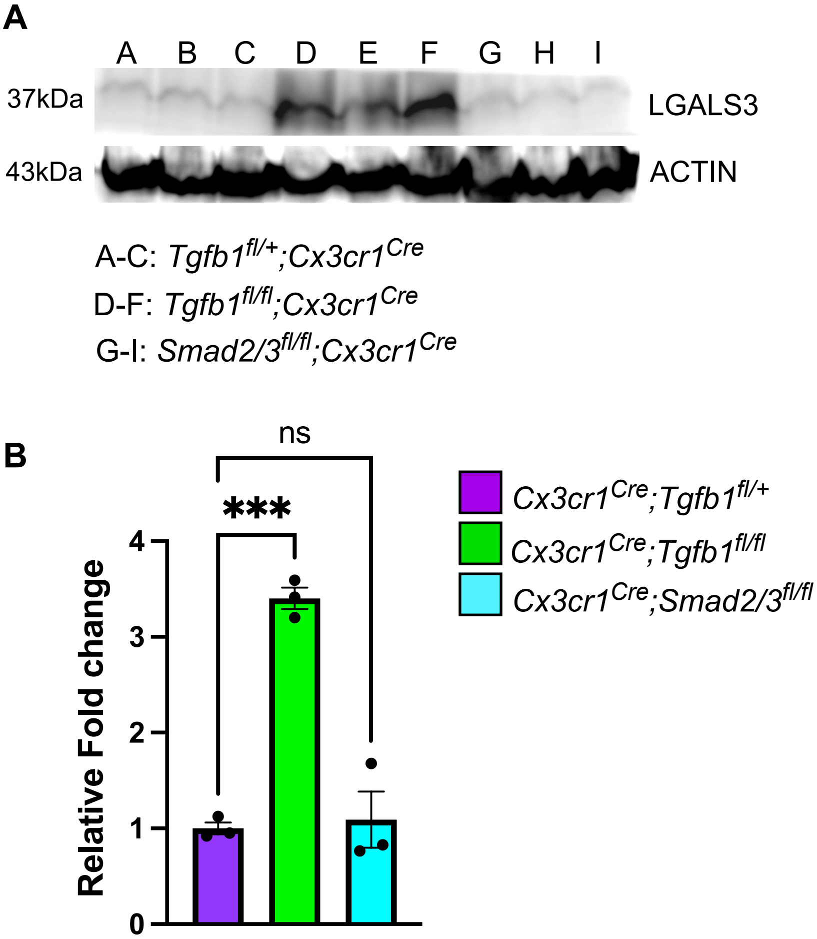

Supplemental Figure 11. Quantification of LGALS3 protein expression in *Tgfb1* mutant models.

A) Western blot measurement of LGALS3 protein expression in whole brain lysates from *Cx3cr1*^{Cre}*;Tgfb1*$^{fl/+}$ controls*; Cx3cr1*^{Cre}*;Tgfb1*$^{fl/+}$ mutants*;* and *Cx3cr1*^{Cre}*;Smad2/3*$^{fl/fl}$ mutant mice. Each well is derived from a separate mouse whole brain lysate. B) Densitometric quantification of Western Blots. Error bars= mean +/- SEM. n=3, ***p<0.001, *n.s.* = not significant (p >0.05), two-tailed unpaired t-test. Source data are provided as a Source Data file.

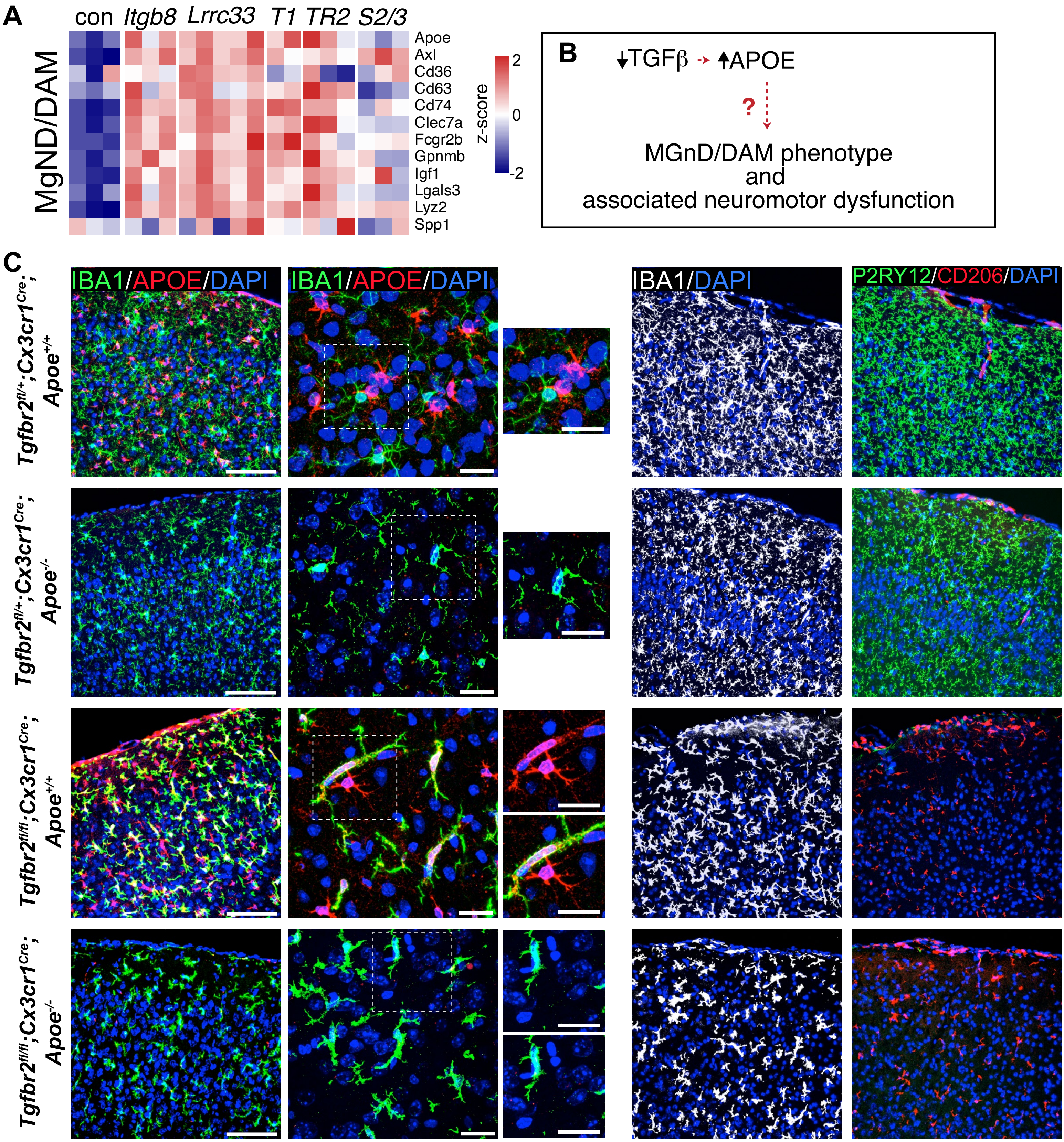

Supplemental Figure 10. Epistatic analysis of *Apoe* contribution to the *Tgfbr2* conditional mutant microglial phenotype.

A) Heatmap of *Apoe* and other MGnD marker gene expression in TGFβ pathway mutants. B) Diagram describing potential pathway interaction between upregulated *Apoe* in the *Tgfbr2* conditional mutant model and the downstream MGnD/DAM microglial phenotype. C) Analysis of effect of simultaneous deletion of *Apoe* and microglial *Tgfbr2*. No change was seen in *Apoe*$^{+/-}$;*Tgfbr2*$^{fl/+}$;*Cx3cr1*^{Cre} *versus Apoe*$^{-/-}$;*Tgfbr2*$^{fl/+}$;*Cx3cr1*^{Cre} in dysmature morphology, P2RY12 expression loss, CD206 upregulation. Scale bar in C=100μm. Scale bar in C=25μm for higher magnification image and insets.

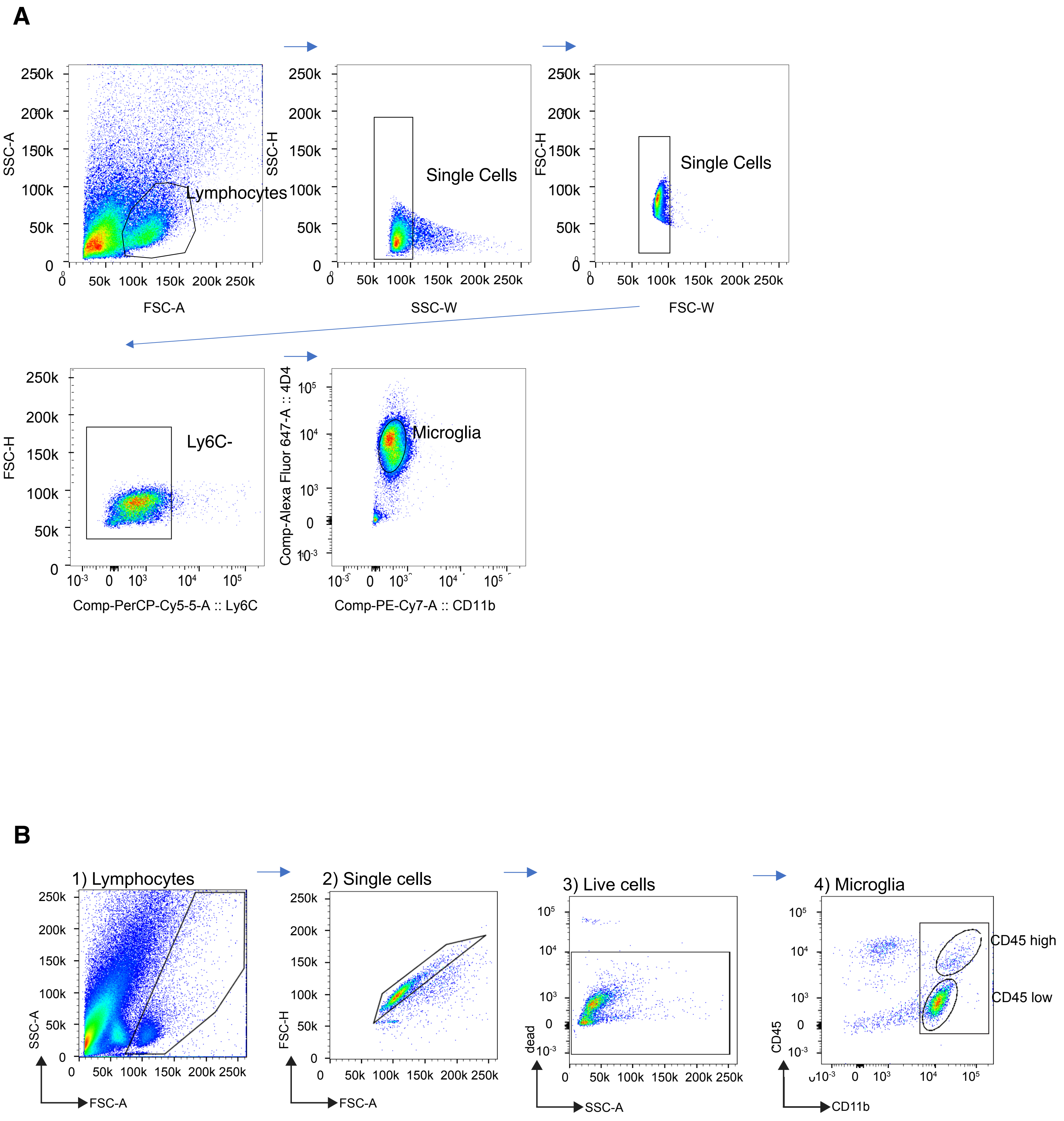

Supplemental Figure 12. Flow gating diagrams.

Representative flow gating method for microglial isolation in Figures 2, 3 (gating strategy A) and Figure S6 and S8 (gating strategy B).
