# Supplementary material for: Radial glia integrin avb8 regulates cell autonomous microglial TGFβ1 signaling that is necessary for microglial identity
Source: Nat Commun. 2025 Mar 22;16:2840. doi: 10.1038/s41467-025-57684-y (PMC11929771; doi:10.1038/s41467-025-57684-y)
Supplement: Supplementary file 2 — Reporting Summary [file 41467_2025_57684_MOESM2_ESM.pdf]

nature portfolio

Corresponding author(s): Gabriel McKinsey, Thomas Arnold

Last updated by author(s): Jan 14, 2024

Reporting Summary

Nature Portfolio wishes to improve the reproducibility of the work that we publish. This form provides structure for consistency and transparency in reporting. For further information on Nature Portfolio policies, see our Editorial Policies and the Editorial Policy Checklist.

Statistics

For all statistical analyses, confirm that the following items are present in the figure legend, table legend, main text, or Methods section.

n/a	Confirmed	
☐	☒	The exact sample size (*n*) for each experimental group/condition, given as a discrete number and unit of measurement
☐	☒	A statement on whether measurements were taken from distinct samples or whether the same sample was measured repeatedly
☐	☒	The statistical test(s) used AND whether they are one- or two-sided<br>*Only common tests should be described solely by name; describe more complex techniques in the Methods section.*
☒	☐	A description of all covariates tested
☐	☒	A description of any assumptions or corrections, such as tests of normality and adjustment for multiple comparisons
☐	☒	A full description of the statistical parameters including central tendency (e.g. means) or other basic estimates (e.g. regression coefficient) AND variation (e.g. standard deviation) or associated estimates of uncertainty (e.g. confidence intervals)
☐	☒	For null hypothesis testing, the test statistic (e.g. *F*, *t*, *r*) with confidence intervals, effect sizes, degrees of freedom and *P* value noted<br>*Give P values as exact values whenever suitable.*
☒	☐	For Bayesian analysis, information on the choice of priors and Markov chain Monte Carlo settings
☒	☐	For hierarchical and complex designs, identification of the appropriate level for tests and full reporting of outcomes
☐	☒	Estimates of effect sizes (e.g. Cohen's *d*, Pearson's *r*), indicating how they were calculated

Our web collection on statistics for biologists contains articles on many of the points above.

Software and code

Policy information about availability of computer code

Data collection

BD FACSDiva was used for collection of samples by FACS.

Data analysis

ImageJ
Graphpad Prism

RNA sequencing: Count normalization and differential expression analysis were performed with DESeq2, considering an FDR<0.05. Correlations between log2FC of genes in multiple datasets were computed using Pearson´s correlation with the cor() function from base R and visualized using the corrplot package. For correlations, we selected genes with differential expression in at least one dataset for a final set of 4406 genes contributing to correlations. Heatmaps were generated with the pheatmap package; units used in each heatmap are indicated in their legend. Correlations between multiple datasets were computed with the cor() function from base R and visualized using the corrplot package. Venn diagrams were produced with standard GNU coreutils and drawn in Inkscape. Overrepresentation analysis was performed using PANTHER from the GO portal. Image analysis and base calling were conducted by the NovaSeq Control Software (NCS). Raw sequence data (.bcl files) generated from Illumina NovaSeq was converted into fastq files and de-multiplexed using Illumina bcl2fastq 2.20 software.

Epigenetic analysis: Raw fastq files were first checked for quality using Multiqc sequence analysis. Cutadapt (v.4.0) was used to cut adaptors (-a AGATCGGAAGAGCACACGTCTGAACTCCAGTC -A AGATCGGAAGAGCGTCGTGTAGGGAAAGAGTGT), and reads were aligned to the mouse genome (mm10) using bowtie2 (2.3.4.3)79. Subsequently, SAM files were sorted and filtered using Sambamba (v.0.8.2). We discarded unmapped and duplicate fragments. Sorted BAM files were indexed using samtools (v.1.15.1). Normalization for all BigWig files were carried out against effective genome size (2652783500 for mice H3K9ac ChIP-seq and ATAC-seq). Peak analysis was carried out with Macs2 (v.2.2.7) using stringent parameters (-f BAMPE --mfold 5 50 -p 0.001). A master-peak file was created, using the following settings for ATAC (-d 300 -c 7) and H3K9ac (d 300 -c 3). Raw counts were extracted from BAM files using master-peak file and loaded into DESeq2. Differentially accessible

peaks were determined with adjustment for false discovery using benjamini hochberg method (Padj < 0.05). Differentially accessible peaks were annotated using HOMER (v4.4)44. BAM files were then converted to BigWig files and merged per experimental group for visualization using deeptools (v.3.5.0). Peak plots were constructed using deeptools with a 1000bp extension from the center of the peak. Peaks were visualized using IGV (v.2.11.4), exported as .png, and edited in Adobe Illustrator.

For manuscripts utilizing custom algorithms or software that are central to the research but not yet described in published literature, software must be made available to editors and reviewers. We strongly encourage code deposition in a community repository (e.g. GitHub). See the Nature Portfolio guidelines for submitting code & software for further information.

Data

Policy information about availability of data

All manuscripts must include a data availability statement. This statement should provide the following information, where applicable:

- Accession codes, unique identifiers, or web links for publicly available datasets
- A description of any restrictions on data availability
- For clinical datasets or third party data, please ensure that the statement adheres to our policy

Provide your data availability statement here.

Research involving human participants, their data, or biological material

Policy information about studies with human participants or human data. See also policy information about sex, gender (identity/presentation), and sexual orientation and race, ethnicity and racism.

Reporting on sex and gender	No human subjects, data, or material were used in this study.
Reporting on race, ethnicity, or other socially relevant groupings	No human subjects, data, or material were used in this study.
Population characteristics	No human subjects, data, or material were used in this study.
Recruitment	No human subjects, data, or material were used in this study.
Ethics oversight	No human subjects, data, or material were used in this study.

Note that full information on the approval of the study protocol must also be provided in the manuscript.

Field-specific reporting

Please select the one below that is the best fit for your research. If you are not sure, read the appropriate sections before making your selection.

☒ Life sciences ☐ Behavioural & social sciences ☐ Ecological, evolutionary & environmental sciences

For a reference copy of the document with all sections, see nature.com/documents/nr-reporting-summary-flat.pdf

Life sciences study design

All studies must disclose on these points even when the disclosure is negative.

Sample size	Sample size was not precalculated using a statistical framework, but conformed to general standards in the field.
Data exclusions	No data has been excluded.
Replication	For all quantification and statistical comparisons, biological replicates were derived from separate mice, with an n of at least 3 for each category.
Randomization	No randomization was used in this study. Samples were allocated into experimental groups according to genotype. Covariates were controlled for by comparing to control genotypes for each experiment.
Blinding	No blinding was used in this study. Gene expression and recombination analysis was done in an automated fashion, so no subjective bias could be introduced.

Reporting for specific materials, systems and methods

We require information from authors about some types of materials, experimental systems and methods used in many studies. Here, indicate whether each material, system or method listed is relevant to your study. If you are not sure if a list item applies to your research, read the appropriate section before selecting a response.

Materials & experimental systems

n/a	Involved in the study
	x Antibodies
x	Eukaryotic cell lines
x	Palaeontology and archaeology
	x Animals and other organisms
x	Clinical data
x	Dual use research of concern
x	Plants

Methods

n/a	Involved in the study
	x ChIP-seq
	x Flow cytometry
x	MRI-based neuroimaging

Antibodies

Antibodies used

Monoclonal: rabbit anti-ApoE (Abcam) (1:300), RRID:AB_2832971; rat anti-CD206 (Biorad)(1:150), RRID:AB_1101333; rat anti-CD11b-PE-Cy7 (eBioscience)(1:300), RRID: AB_469588; rat anti-Clec7a (BioLegend) (1:300), RRID:AB_2561519; rat anti-Fcrls-APC (Butovsky Lab) (1:1000), Clone 4G11; anti IB4 Alexa fluor 488 conjugate (Thermo Fisher) (1:150), RRID:AB_2314662; rat anti-LGALS3 (CedarLane) (1:150), RRID:AB_10060357; rat anti-Ly-6C-PerCP/Cy5.5 (Biolegend) (1:300), RRID:AB_1659241; mouse anti-Nestin-488 (Abcam) (1:150), RRID:AB_2732861; mouse anti-NeuN (Millipore) (1:100), RRID:AB_2298772; rabbit anti-pSmad3 (S423/S425) (Abcam) (1:100), RRID: AB_882596; rabbit anti-Tmem119 (Abcam) (1:100), RRID:AB_2800343.

Polyclonal: goat anti-CD31 (R&D Systems) (1:300), RRID: AB_2161028; goat anti-EGFP (Origene) (1:2000), RRID:AB_1002036; goat anti-IBA1 (Novus) (1:300), RRID:AB_521594; rabbit anti-IBA1 (Wako) (1:300), RRID:AB_521594; rabbit anti-Olig2-Rb (Millipore) (1:300), RRID:AB_570666; rabbit anti-P2ry12 (Anaspec) (1:300), RRID:AB_2298886; rabbit anti-P2ry12 (David Julius) (1:1000); goat anti-PdgfRa (R&D Systems) (1:300), RRID:AB_2236897; rabbit anti-GFAP (DAKO) (1:300), RRID:AB_10013382; goat anti-Sox9 (R&D Systems) (1:300), RRID:AB_2194160; rat anti-Ter119 (R&D Systems) (1:150), RRID: AB_2297123.

Validation

Validation: Rabbit anti-ApoE (Abcam) validated by manufacturer with 17 citations (https://www.abcam.com/en-us/products/primary-antibodies/apolipoprotein-e-antibody-epr19378-ab183596?srsltid=AfmBOoqINbY341ASntcAcoU6hfW4CF6eB5WWXJ7NhZ8I5V9I5Qk2JTPK). Rat anti-CD206 (Biorad) validated by manufacturer with 91 citations (https://www.bio-rad-antibodies.com/monoclonal/mouse-cd206-antibody-mr5d3-mca2235.html?f=purified). Rat anti-CD11b-PE-Cy7 (eBioscience) validated by manufacturer for flow cytometry (https://www.fishersci.com/shop/products/cd11b-monoclonal-antibody-m1-70-pe-cyanine7-ebioscience-invitrogen/5015454). Rat anti mouse CD45-APC (BD Biosciences) validated by manufacturer with 272 citations (https://www.bdbiosciences.com/en-us/products/reagents/flow-cytometry-reagents/research-reagents/single-color-antibodies-ruo/apc-rat-anti-mouse-cd45.559864?tab=product_details). Goat anti-CD31 (R&D Systems) validated by manufacturer with 14 citations (https://www.rndsystems.com/products/human-mouse-rat-cd31-pecam-1-antibody_af3628). Rat anti-Clec7a (BioLegend) validated by manufacturer with 2 citations (https://www.biolegend.com/de-at/products/purified-anti-mouse-cd369-dectin-1-clec7a-antibody-8146). Goat anti-EGFP (Origene) validated by manufacturer with 22 citations (https://www.origene.com/catalog/antibodies/primary-antibodies/r1091p-gfp-ads-to-hu-ms-rt-serum-proteins-goat-polyclonal-antibody). Anti IB4 Alexa fluor 488 conjugate (Thermo Fisher) validated by manufacturer with 39 citations (https://www.thermofisher.com/order/catalog/product/I21411). Goat anti-IBA1 (Novus) validated by manufacturer with 379 citations (https://www.novusbio.com/products/aif-1-iba1-antibody_nb100-1028?srsltid=AfmBOoreYIeznhy8G1pSwQzCuiRkveEf74hlUMgFvlvw16aCsoXrUkBC). Rabbit anti-IBA1 (WAKO) validated by manufacturer with 4,136 citations (https://labchem-wako.fujifilm.com/us/product/detail/W01W0101-1974.html). Rat anti-LGALS3 (CedarLane) validated by manufacturer with over 11 citations (https://www.cedarlanelabs.com/Products/Detail?code=CL8942AP&gaclick=True&lob=AllProducts). Rat anti-Ly-6C-PerCP/Cy5.5 (Biolegend) validated by manufacturer with 107 citations (https://www.biolegend.com/de-at/clone-search/percp-cyanine5-5-anti-mouse-ly-6c-antibody-5967). Mouse anti-Nestin-488 (Abcam) validated by manufacturer with 1 citation (https://www.abcam.com/en-us/products/primary-antibodies/alexa-fluor-488-nestin-antibody-rat-401-ab197495?srsltid=AfmBOoqPztwyaj_VOhfgPXasGywTxsNp9t_SfFr1owqkmdGJsUxYJ_l5). Mouse anti-NeuN (Millipore) validated by manufacturer with over 1,000 citations (https://www.emdmillipore.com/US/en/product/Anti-NeuN-Antibody-clone-A60,MM_NF-MAB377). Rabbit anti-Olig2-Rb (Millipore) validated by manufacturer with over 100 citations (https://www.emdmillipore.com/US/en/product/Anti-Olig-2-Antibody,MM_NF-AB9610). Rabbit anti-P2ry12 (Anaspec) validated by manufacturer with over 20 citations. https://www.bioz.com/result/rabbit%20anti%20p2y12/product/AnaSpec. Goat anti-PdgfRa (R&D Systems) validated by manufacturer with 140 citations (https://www.rndsystems.com/products/mouse-pdgf-ralpha-antibody_af1062). Rabbit anti-GFAP (DAKO) validated by manufacturer with 1 citation. https://www.agilent.com/store/productDetail.jsp?catalogId=Z033429-2. Rabbit anti-pSmad3 (S423/S425) (Abcam) validated by manufacturer with over 500 citations (https://www.abcam.com/en-us/products/primary-antibodies/smad3-ps423-425-smad5-ps463-465-smad1-ps463-465-smad2-ps465-467-antibody-ep823y-ab52903?srsltid=AfmBOopvDeUJSwHrM5X70bnEzxyaiGBCJj8eUWOU_caXM2GzwcJICoLh). Goat anti-Sox9 (R&D Systems) validated by manufacturer with 80 citations (https://www.rndsystems.com/products/human-sox9-antibody_af3075). Rat anti-Ter119 (R&D Systems) validated by manufacturer with over 10 citations (https://www.rndsystems.com/products/mouse-ter-119-antibody-ter-119_mab1125). Rabbit anti-Tmem119 (Abcam) validated by manufacturer with over 100 citations (https://www.abcam.com/en-us/products/primary-antibodies/tmem119-antibody-28-3-microglial-marker-ab209064?srsltid=AfmBOoqO0I4He351BnSb8fMwxllUU6U4Y61vdtJkIboGRGmWuv7o5wb3).

Animals and other research organisms

Policy information about studies involving animals; ARRIVE guidelines recommended for reporting animal research, and Sex and Gender in Research

Laboratory animals	We used Mus musculus in this study. All mice were backcrossed to C57BL6/J, except for Tgfb1 constitutive null mice, which were on the NIH/OlaHsd background. Mice were analyzed at E14.5, P30 and P60-90.
Wild animals	Not applicable.
Reporting on sex	Findings applied to both sexes. Sex-dependent differences were not examined explicitly, as it was not a primary variable of interest, with the exception of the histological analysis of Lrrc33 mutants.
Field-collected samples	Not applicable.
Ethics oversight	All animal studies were followed by the protocol approved by the Institutional Animal Care and Use Committee (IACUC) at UCSF, protocol #AN194997.

Note that full information on the approval of the study protocol must also be provided in the manuscript.

Plants

Seed stocks	No plants were used in this study.
Novel plant genotypes	No plants were used in this study.
Authentication	No plants were used in this study.

ChIP-seq

Data deposition

- [x] Confirm that both raw and final processed data have been deposited in a public database such as GEO.
- [x] Confirm that you have deposited or provided access to graph files (e.g. BED files) for the called peaks.

Data access links<br>*May remain private before publication.*	The sequencing data generated in this study have been deposited in the GEO database under accession codes as follows: ChIP-seq data have been deposited with the accession code GSE242221 (https://www.ncbi.nlm.nih.gov/geo/query/acc.cgi?acc=GSE242221).(RNA-seq data (whole brain data, isolated Tgfb1 mutant microglia datasets) have been deposited with the accession GSE236615 (https://www.ncbi.nlm.nih.gov/geo/query/acc.cgi?acc=GSE236615). Public ATAC-seq data was obtained from GSE79816 (https://www.ncbi.nlm.nih.gov/geo/query/acc.cgi?acc=GSE79816). Itgb8fl/fl;Emx1Cre microglia and Smad2/3fl/fl;Cx3cr1Cre data were generated previously by our group and are available under GEO GSE239603 (https://www.ncbi.nlm.nih.gov/geo/query/acc.cgi?acc=GSE239603). Tgfbr2fl/fl;Cx3cr1Cre microglia is from GEO GSE124868 (https://www.ncbi.nlm.nih.gov/geo/query/acc.cgi?acc=GSE12468). Data from Lrrc33-/- microglia and whole brain was obtained from GEO GSE96938. (https://www.ncbi.nlm.nih.gov/geo/query/acc.cgi?acc=GSE96938).
Files in database submission	Raw fastq files and processed bigwig files.
Genome browser session<br>(e.g. UCSC)	http://genome.ucsc.edu/s/Gabriel_McKinsey/McKinsey%20et%20al.%20ATAC%2Dseq%20and%20H3K9ac%20data

Methodology

Replicates	N=7 for WT and ITGB8-KO ATAC-seq. N=4 for WT and N=3 for ITGB8-KO H3K9ac ChIP-seq (7 matched input controls for H3K9ac).
Sequencing depth	10-15 million reads per sample.
Antibodies	H3K9ac: Millipore 07-352.
Peak calling parameters	Stringent MACS2 parameters: -f BAMPE --mfold 5 50 -p 0.001.
Data quality	Exceptional data quality: 50,000 highly reproducible peaks in ATAC-seq and 25,000 in H3K9ac ChIP-seq.
Software	Cutadapt (v.4.0), bowtie2 (2.3.4.3), Sambamba (v.0.8.2), samtools (v.1.15.1), Macs2 (v.2.2.7), deeptools (v.3.5.0), HOMER (v4.4).

Flow Cytometry

Plots

Confirm that:

- [ ] The axis labels state the marker and fluorochrome used (e.g. CD4-FITC).
- [ ] The axis scales are clearly visible. Include numbers along axes only for bottom left plot of group (a 'group' is an analysis of identical markers).
- [ ] All plots are contour plots with outliers or pseudocolor plots.
- [ ] A numerical value for number of cells or percentage (with statistics) is provided.

Methodology

Sample preparation	The flow procedure was only used for cell sorting and subsequent transcriptional analysis. Flow plots are not present in the manuscript as flow cytometry itself was not used for analysis.<br><br>The Itgb8fl/fl;Emx1Cre Mice were euthanized in a CO_2 chamber and then transcardially perfused with 10 ml cold Hanks' Balanced Salt Solution (HBSS, ThermoFisher, 14175103). The mouse brain was isolated and the cortex was dissected from one hemisphere for microglia purification using the standardard isolation procedure established in Butovsky lab58 Briefly, the cortex was homogenized and resuspended with 5 ml 70% Percoll Plus (GE Healthcare, 17-5445-02) and 5 ml 37% Percoll Plus placed on top. The microglia were enriched in the interface layer after centrifugation in 800 g, 4°C, for 25 min with an acceleration of 2 and a deceleration of 1. The microglia enriched cell population were stained with PE-Cy7 anti-mouse CD11b (1:300, eBioscience, 50-154-54), APC anti-mouse Fcrls (1:1000, clone 4G11, Butovsky Lab), and PerCP/Cy5.5 anti-mouse Ly-6C (1:300, Biolegend, 128012). The cells were then processed by a BD FACSAriaTM II (BD Bioscience) and CD11b+Fcrls+ Ly-6C– cells were sorted into Eppendorf tubes for RNA-seq. Microglia from Smad2/3fl/fl;Cx3cr1Cre and Tgfb1fl/fl;Cx3cr1Cre mice were isolated using a percol gradient isolation strategy. To isolate single cells, brains were cut into small pieces and passed through a 40 µm filter. Single cell suspensions were prepared and centrifuged over a 30%/70% discontinuous Percoll gradient (GE Healthcare) and mononuclear cells were isolated from the interface. Flow cytometry was performed on a FACS Aria III using the FACSDiva 8.0 software (BD Biosciences). .
Instrument	BD FACS AriaTm II.
Software	BD FACSDiva.
Cell population abundance	Final CD45 and CD11b positive cells were ~10% of total singlets.
Gating strategy	Microglia were isolated by sorting for CD45 and CD11b positive cells following initial gating for forward scatter, singlets and live cells.

- [ ] Tick this box to confirm that a figure exemplifying the gating strategy is provided in the Supplementary Information.
